# Relationship between nutritional status and length of hospital stay among patients with atrial fibrillation – a result of the nutritional status heart study

**DOI:** 10.3389/fnut.2022.1086715

**Published:** 2022-12-14

**Authors:** Michał Czapla, Izabella Uchmanowicz, Raúl Juárez-Vela, Angela Durante, Marta Kałużna-Oleksy, Katarzyna Łokieć, Ramón Baeza-Trinidad, Jacek Smereka

**Affiliations:** ^1^Department of Emergency Medical Service, Wroclaw Medical University, Wrocław, Poland; ^2^Institute of Heart Diseases, University Hospital, Wrocław, Poland; ^3^Group of Research in Care (GRUPAC), Faculty of Health Sciences, University of La Rioja, Logroño, Spain; ^4^Department of Nursing and Obstetrics, Faculty of Health Sciences, Wroclaw Medical University, Wrocław, Poland; ^5^1st Department of Cardiology, Poznań University of Medical Sciences, Poznań, Poland; ^6^Department of Propaedeutic of Civilization Diseases, Medical University of Lodz, Lodz, Poland; ^7^Internal Medicine Department, San Pedro Hospital, Logroño, Spain

**Keywords:** nutritional status, malnutrition, obesity, atrial fibrillation, length of hospital stay, BMI

## Abstract

**Background:**

Nutritional status is related to the prognosis and length of hospital stay (LOHS) of patients with atrial fibrillation (AF). This study aimed to assess how nutritional status affects LOHS for patients with AF.

**Methods:**

We performed retrospective analysis of the medical records of 1,813 patients admitted urgently with a diagnosis of AF to the Institute of Heart Diseases of the University Clinical Hospital in Wroclaw, Poland.

**Results:**

In total, 1,813 patients were included in the analysis. The average LOHS in the entire group was 3.53 ± 3.41 days. The mean BMI was 28.7 kg/m^2^ (SD: 5.02). Patients who were hospitalized longer were statistically more likely to have a Nutritional Risk Score (NRS) ≥3 (*p* = 0.028). A higher percentage of longer hospitalized patients with LDL levels below 70 mg/dl (*p* < 0.001) and those with HDL ≥40 mg/dl (*p* < 0.001) were observed. Study participants with NRS ≥3 were an older group (*M* = 76.3 years), with longer mean LOHS (*M* = 4.44 days). The predictors of LOHS in the univariate model were age (OR = 1.04), LDL (OR = 0.99), HDL (OR = 0.98), TC (OR = 0.996), CRP (OR = 1, 02, *p* < 0.001), lymphocytes (OR = 0.97, *p* = 0.008) and in the multivariate model were age, LDL (mg/dl), HDL (mg/dl), Na, and K.

**Conclusion:**

For nutritional status, factors indicating the risk of prolonged hospitalization in patients with AF are malnutrition, lower serum LDL, HDL, potassium, and sodium levels identified at the time of admission to the cardiology department. Assessment of nutritional status in patients with AF is important both in the context of evaluating obesity and malnutrition status, as both conditions can alter the prognosis of patients. Further studies are needed to determine the exact impact of the above on the risk of prolonged hospitalization.

## 1 Introduction

Atrial fibrillation (AF) is the most common heart rhythm disorder worldwide. AF deaths in 2017 totaled 287,200 worldwide (1). As many as 37.6 million cases of AF were reported in 2017, and the Global Burden of Disease Project estimated that the size of the AF patient population in 2016 was 46.3 million (2). Data from the Framingham Heart Study (FHS) indicated that the incidence of AF has increased threefold over the past 50 years (3). However, it should be remembered that one third of the total AF population is asymptomatic, making the data presented here certainly an underestimate (4). The reasons for the increase in the number of patients with AF include an aging population, longer survival times for those with heart conditions (e.g., ischemic heart disease, cardiomyopathies, and heart failure) that predispose to AF, and the increasing prevalence of risk factors that include obesity and hypertension (5, 6). In new AF diagnosis, the prevalence of comorbidities, including obesity, hypertension, diabetes, and chronic lung disease, increased over time (7, 8). It is believed that screening advanced-age patients with AF for nutritional disorders may be beneficial for the early detection of patients at high risk of adverse clinical outcomes, thereby improving their prognosis (9).

According to the research, obesity gradually increases the risk of AF, and depending on body mass index (BMI), it can also increase the risk of ischemic stroke, thromboembolic complications and death. Although there is a phenomenon called the “obesity paradox” among AF patients concerning all-cause and cardiovascular deaths, there is an opposite relationship between overweight/obesity and better cardiovascular prognosis in long-term follow-up. Therefore, it is essential to assess the nutritional status of this group of patients (10–12). Poor nutritional status of the patient is also a reason for prolonged length of hospital stay (LOHS) (13). When a patient is malnourished, the risk of complications increases, the effectiveness of treatment decreases and the cost of hospitalization increases (14). Consequently, it is necessary to properly assess the patient’s nutritional status on admission to the hospital (14). In Poland, under current legislation, a screening tool such as the Nutritional Risk Score 2002 (NRS 2002) or Subjective Global Assessment (SGA) is used to assess nutritional status during hospital admission, which is in line with the Global Leadership Initiative on Malnutrition guidelines (GLIM) (15).

This study aims to evaluate the impact of nutritional status upon admission to the hospital on the LOHS of patients with atrial fibrillation.

## 2 Materials and methods

### 2.1 Study design and setting

A retrospective analysis of the medical records of 1,813 patients admitted urgently with a diagnosis of AF (ICD10: I48) to the Institute of Heart Diseases (University Clinical Hospital in Wroclaw, Poland) between January 2017 and June 2021 was conducted.

### 2.2 Study population and data

Record analysis was performed on all patients who met the inclusion criteria, i.e., a diagnosis of AF (primary reason for hospital admission) and age older than 18 years old. Data from 1,813 medical records were analyzed, on categories such as sex, age, classification of AF-related symptoms (EHRA score), and type of AF (paroxysmal, persistent, and permanent) (16), BMI (kg/m^2^) score, nutritional risk by NRS 2002 and LOHS. In comorbidities, the categories studied were diabetes mellitus (DM), heart failure (HF), hypertension (HT), chronic kidney disease (CKD), acute coronary syndrome (ACS), cerebral stroke (CS), hyperthyroidism, and hypothyroidism. Analysis was conducted on laboratory test results such as N-terminal pro-B type natriuretic peptide (NT-proBNP), triglycerides (TGs), high-density lipoprotein (HDL), low-density lipoprotein (LDL), total cholesterol (TC), C-reactive protein (CRP), procalcitonin (PCT), transferrin, albumins, lymphocytes, potassium (K), sodium (Na), thyroid-stimulating hormone (TSH), free triiodothyronine (FT3), free thyroxine (FT4), and hemoglobin A1c (HbA1c). Blood for laboratory tests was drawn by a nurse at the time of admission to the cardiology department. The doctor admitting the patient to the cardiology department decided which test the patient had. The tests were performed in the hospital’s laboratory (Alinity C, Abbott) in accordance with the principles of Good Laboratory Practice.

### 2.3 Nutritional screening

Nutritional status was assessed using NRS 2002. This tool is based on disease severity, impaired nutritional status (BMI, weight loss, and food intake in the preceding week) and age. A patient can obtain a score of 0 to 7 points. A score of ≥3 indicates that a patient is at nutritional risk (17). Criteria for assessing BMI were used according to the guidelines Word Health Organization (WHO), i.e., underweight (BMI <18.5), normal body mass (BMI 18.5–24.9), pre-obese (BMI 25–29.9), and obese (BMI ≥30) (18). The NRS 2002 and BMI were assessed by the physician admitting the patient to the cardiology department.

### 2.4 Ethical considerations

The study was conducted following the principles of the Declaration of Helsinki and approved by the independent Bioethics Committee of Wroclaw Medical University, protocol no. KB-205/2021. The study followed the STROBE guidelines (Strengthening the Reporting of Observational Studies in Epidemiology).

### 2.5 Statistical analysis

For measurable variables, means, medians, quartiles, minimum and maximum values, and standard deviations were calculated. Due to the lack of data on some parameters, these numbers are smaller and provide for each variable. All quantitative-type variables studied were checked using the Shapiro–Wilk test to determine the type of distribution. Inter-group comparisons of the results of quantitative variables were carried out using the *t*-test or Mann–Whitney U test (depending on whether the assumptions were met). A comparison of the results of more than two groups was carried out using a one-way analysis of variance or the Kruskal–Wallis test (depending on whether the assumptions were met). A Chi-squared test or Fisher’s exact test was used to compare qualitative variables. In addition, an analysis of the effect of selected factors on the LOHS (days) was performed using linear regression (a univariate model of the predictors included in the analysis). The unstandardized and standardized regression coefficient, standard error, and level of statistical significance were determined. The next step was to build a multivariate model (stepwise progressive method), considering variables whose *p*-value in the univariate model was less than or equal to 0.3. An analysis of the effect of selected factors on the LOHS categorized as <5 days and ≥5 (≥75th centile) was also performed using logistic regression (univariate model of predictors included in the analysis). Odds quotients and confidence intervals were determined. The next step was to build a multivariate model. The model-building process was carried out using progressive stepwise regression and a set of standard measures of goodness of fit (AIC, BIC, and Hosmer–Lemeshow test) was used to evaluate the model. Statistical analysis was performed using Statistica 13.1 software (Tibco, Inc., Palo Alto, CA, USA).

## 3 Results

### 3.1 Group comparison

The characteristics of the entire group and the comparison of these characteristics between the group of patients who were hospitalized for less than 5 days and for 5 days or more are shown in Table 1 (qualitative and quantitative variables). A total of 1,813 people were included in the analysis. The average LOHS in the entire group was 3.53 ± 3.41 days. Statistically significant differences between groups were noted when comparing against sex, type of AF, EHRA class, NRS, DM, CS, HT, LDL, or HDL levels. Patients who were hospitalized longer were statistically more likely to have: permanent AF (24% vs. 16%; *p* < 0.001), EHRA class III (64% vs. 48%; *p* < 0.001), NRS ≥3 (10 vs. 6%; *p* = 0.028). The following were not established: DM (82 vs. 78%; *p* = 0.037), CS (93 vs. 87%; *p* = 0.001), and HT (56 vs. 39%; *p* < 0.001). In addition, a higher percentage of longer hospitalized patients with LDL levels below 70 mg/dl (28 vs. 21%; *p* < 0.001) and those with HDL ≥40 mg/dl (35 vs. 23%; *p* < 0.001) were observed. For quantitative variables, the age of patients hospitalized for longer than 5 days was statistically higher. In addition, this group had statistically significant higher results for parameters such as CRP, BNP, and HbA1c. Statistically lower results in this group were for variables such as LDL, HDL, TC, lymphocytes, K, and Na (Table 1). Due to the lack of data on some parameters, these numbers are smaller and provide for each variable.

**TABLE 1:** Study group characteristics and comparison of quantitative and qualitative variables relative to length of hospital stay.

Parameter		Total (*N* = 1,813) *N* (%)	Length of hospital stay (days)		*P*-value*
			
			<5	≥5	
Sex	Female	791 (44%)	552 (42%)	239 (49%)	0.006*
	Male	1,022 (56%)	772 (58%)	250 (51%)	
BMI (kg/m^2^)	18.5–24.9	328 (25%)	238 (23%)	90 (27%)	0.513
	25.0–29.9	502 (37%)	380 (38%)	122 (36%)	
	≥30.0	512 (38%)	388 (39%)	124 (37%)	
Type of AF	Paroxysmal	702 (39%)	528 (40%)	174 (36%)	<0.001*
	Persistent	783 (43%)	588 (44%)	195 (40%)	
	Permanent	328 (18%)	208 (16%)	120 (24%)	
EHRA AF	I	33 (6%)	29 (7%)	4 (4%)	0.005*
	IIa	239 (27%)	107 (26%)	32 (30%)	
	IIb	169 (32%)	148 (36%)	21 (19%)	
	III	174 (33%)	125 (30%)	49 (45%)	
	IV	9 (2%)	7 (2%)	2 (2%)	
NRS	<3	1,484 (93%)	1,091 (94%)	393 (90%)	0.028*
	≥3	117 (7%)	75 (6%)	42 (10%)	
HF	No	1,478 (82%)	1,080 (82%)	398 (81%)	0.930
	Yes	335 (18%)	244 (18%)	91 (19%)	
DM	No	1,431 (79%)	1,029 (78%)	402 (82%)	0.037*
	Yes	382 (21%)	295 (22%)	87 (18%)	
CKD	No	1,569 (87%)	1,156 (87%)	413 (84%)	0.114
	Yes	244 (13%)	168 (13%)	76 (16%)	
CS	No	1,609 (89%)	1,156 (87%)	453 (93%)	0.001*
	Yes	204 (11%)	168 (13%)	36 (7%)	
HT	No	783 (43%)	511 (39%)	272 (56%)	<0.001*
	Yes	1,030 (57%)	813 (61%)	217 (44%)	
ACS	No	1,627 (90%)	1,183 (89%)	444 (91%)	0.367
	Yes	186 (10%)	141 (11%)	45 (9%)	
TD	No	1,461 (81%)	1,083 (82%)	378 (77%)	0.066
	Hyperthyroidism	125 (7%)	82 (6%)	43 (9%)	
	Hypothyroidism	227 (12%)	159 (12%)	68 (14%)	
TG	<135 mg/dl	1,135 (67%)	816 (66%)	319 (68%)	0.805
	135–200 mg/dl	416 (24%)	306 (25%)	110 (23%)	
	>200 mg/dl	153 (9%)	110 (9%)	43 (9%)	
LDL	<70 mg/dl	394 (23%)	262 (21%)	132 (28%)	<0.001*
	70–116 mg/dl	681 (40%)	464 (38%)	217 (46%)	
	>116 mg/dl	624 (37%)	501 (41%)	123 (26%)	
HDL	<40 mg/dl	446 (26%)	282 (23%)	164 (35%)	<0.001*
	≥40 mg/dl	1,256 (74%)	947 (77%)	309 (65%)	
Age (years)	Mean ± SD	68.72 ± 12.09	67.37 ± 12.12	72.35 ± 11.25	<0.001*
	Median	70.00	69.00	72.00	
	Quartiles	63.00–76.00	62.00–74.00	66.00–81.00	
NRS (points)	Mean ± SD	0.94 ± 0.95	0.92 ± 0.94	1.00 ± 0.96	0.122
	Median	1.00	1.00	1.00	
	Quartiles	0.00–2.00	0.00–2.00	0.00–1.00	
BMI (kg/m^2^)	Mean ± SD	28.78 ± 5.02	28.82 ± 4.98	28.70 ± 5.12	0.706
	Median	28.30	28.30	28.10	
	Quartiles	25.00–32.00	25.10–32.00	24.70–31.75	
TG (mg/dl)	Mean ± SD	124.17 ± 61.38	124.38 ± 61.09	123.62 ± 62.19	0.818
	Median	110.00	111.00	108.00	
	Quartiles	83.00–149.00	82.50–149.50	83.50–147.00	
LDL (mg/dl)	Mean ± SD	106.20 ± 45.57	110.23 ± 47.08	95.72 ± 39.55	<0.001*
	Median	97.00	102.00	87.00	
	Quartiles	71.00–134.00	73.00–141.00	67.50–118.00	
HDL (mg/dl)	Mean ± SD	48.57 ± 13.18	49.59 ± 13.06	45.91 ± 13.15	<0.001*
	Median	47.00	48.00	44.00	
	Quartiles	39.00–57.00	40.00–57.00	37.00–55.00	
TC (mg/dl)	Mean ± SD	167.68 ± 61.38	169.91 ± 46.50	161.89 ± 42.71	0.001*
	Median	159.00	161.00	155.00	
	Quartiles	135.00–197.00	136.00–199.00	132.00–188.00	
CRP (mg/L)	Mean ± SD	7.06 ± 22.15	4.77 ± 17.18	12.04 ± 29.68	<0.001*
	Median	1.77	1.58	2.66	
	Quartiles	0.93–4.38	0.85–3.42	1.15–7.78	
Albumin (g/dl)	Mean ± SD	3.46 ± 0.58	3.35 ± 0.67	3.52 ± 0.52	0.160
	Median	3.60	3.50	3.60	
	Quartiles	3.20–3.90	2.85–3.80	3.30–3.90	
Transferrin (g/L)	Mean ± SD	2.54 ± 0.62	2.57 ± 0.60	2.52 ± 0.64	0.598
	Median	2.49	2.48	2.50	
	Quartiles	2.14–2.89	2.16–2.87	2.11–2.90	
Lymphocytes (%)	Mean ± SD	26.42 ± 9.26	27.37 ± 8.54	24.97 ± 10.13	0.007*
	Median	26.75	27.40	24.45	
	Quartiles	19.70–32.80	22.20–32.30	17.35–33.10	
PCT (ng/ml)	Mean ± SD	0.68 ± 4.23	0.13 ± 0.21	0.89 ± 4.96	0.313
	Median	0.08	0.09	0.08	
	Quartiles	0.03–0.18	0.03–0.15	0.03–0.22	
TSH (uIU/ml)	Mean ± SD	1.65 ± 1.28	1.62 ± 1.24	1.73 ± 1.37	0.107
	Median	1.32	1.32	1.31	
	Quartiles	0.88–2.06	0.88–2.02	0.87–2.13	
FT3 (pg/ml)	Mean ± SD	2.98 ± 1.22	2.93 ± 0.60	3.04 ± 1.74	0.538
	Median	2.90	2.96	2.86	
	Quartiles	2.57–3.19	2.61–3.24	2.52–3.13	
FT4 (ng/dl)	Mean ± SD	1.43 ± 1.15	1.46 ± 1.43	1.38 ± 0.54	0.622
	Median	1.33	1.32	1.34	
	Quartiles	1.13–1.54	1.14–1.53	1.09–1.57	
BNP (pg/ml)	Mean ± SD	353.81 ± 563.52	294.67 ± 440.11	420.72 ± 671.35	0.014*
	Median	200.45	159.55	243.50	
	Quartiles	94.70–387.00	67.20–328.80	124.95–459.10	
NT-proBNP	Mean ± SD	2,180.59 ± 4,561.61	1,827.13 ± 4,275.26	3,836.27 ± 5,434.93	<0.001*
	Median	931.75	816.60	2,315.00	
	Quartiles	341.00–2,183.55	307.60–1,754.00	678.80–4,268.80	
K (mmol/L)	Mean ± SD	4.38 ± 0.47	4.40 ± 0.43	4.33 ± 0.57	0.002*
	Median	4.37	4.40	4.29	
	Quartiles	4.09–4.63	4.11–4.66	4.00–4.59	
Na (mmol/L)	Mean ± SD	140.09 ± 3.07	140.30 ± 2.75	139.54 ± 3.72	<0.001*
	Median	140.00	141.00	140.00	
	Quartiles	139.00–142.00	139.00–142.00	138.00–142.00	
HbA1c (%)	Mean ± SD	6.17 ± 0.91	6.13 ± 0.84	6.26 ± 1.05	0.026*
	Median	6.00	6.00	6.00	
	Quartiles	5.60–6.50	5.60–6.40	5.60–6.50	

LOHS, length of hospital stay; N, number of patients; AF, atrial fibrillation; EHRA, European Heart Rhythm Association; NRS, Nutritional Risk Score 2002; BMI, body mass index; HF, heart failure; DM, diabetes mellitus; CKD, chronic kidney disease; CS, cerebral stroke; HT, hypertension; ACS, acute coronary syndrome; TD, thyroid disease; TG, triglycerides; LDL, low-density lipoprotein; HDL, high-density lipoprotein; TC, total cholesterol; CRP, C-reactive protein; PCT, procalcitonin; TSH, thyroid-stimulating hormone; FT3, free triiodothyronine; FT4, free thyroxine; BNP, brain natriuretic peptide; K, potassium; Na, sodium; HbA1c, hemoglobin A1c; p, t-test or Mann–Whitney U test for quantitative variables, Chi-squared or Fisher’s exact test for qualitative variables.

*Statistically significant (*p* < 0.05).

### 3.2 Characteristics of the study group by BMI (kg/m^2^)

A comparison of the evaluated variables between groups according to BMI (kg/m^2^) is shown in Table 2. Based on BMI score, three groups were identified: normal (18.5–24.9 kg/m^2^), overweight (25.0–29.9 kg/m^2^), and obese (≥30 kg/m^2^) subjects. In the study group, there were no patients with a BMI less than 18.5 kg/m^2^. Statistically differences were found by age, TG, HDL, lymphocyte, BNP, TSH, or HbA1c levels, and by type of AF, NRS, DM, CKD, CS, and HT. Participants in the study with BMI ≥30 were the youngest group (*M* = 66.5 years), with the highest levels of TG (*M* = 138.2 mg/dl) and HbA1c (*M* = 6.26%), the lowest levels of HDL (*M* = 46.33 mg/dl), BNP (*M* = 228.8 pg/ml) relative to the other groups. This group also had the highest percentage of subjects with NRS >3 score (98%), persistent AF (56%), DM (25%), HT (61%), TG levels 135–200 mg/dl (29%), and >200 mg/dl (13%) and HDL levels <40 mg/dl (31%). In addition, the lowest percentages of CKD (11%), CS (9%) were observed in this group (Table 2).

**TABLE 2:** Comparison of the assessed parameters by BMI status (qualitative and quantitative variables).

Parameter		BMI (kg/m^2^)			*P*-value
		
		18.5–24.9328 (24%)	25.0–29.9502 (37%)	≥30512 (38%)	
Sex	Female	153 (47%)	194 (39%)	221 (43%)	0.066
	Male	175 (53%)	308 (61%)	291 (57%)	
NRS	<3	262 (83%)	416 (93%)	434 (98%)	<0.001*
	≥3	55 (17%)	31 (7%)	7 (2%)	
Type of AF	Paroxysmal	146 (45%)	213 (42%)	158 (31%)	<0.001*
	Persistent	109 (33%)	194 (39%)	287 (56%)	
	Permanent	73 (22%)	95 (19%)	67 (13%)	
EHRA class	I	6 (9%)	4 (3%)	16 (9%)	0.009
	IIa	13 (19%)	37 (26%)	57 (32%)	
	IIb	24 (34%)	55 (39%)	53 (30%)	
	III	23 (33%)	45 (32%)	50 (28%)	
	IV	4 (6%)	1 (1%)	1 (1%)	
HF	No	267 (81%)	421 (84%)	400 (78%)	0.065
	Yes	61 (19%)	81 (16%)	112 (22%)	
DM	No	280 (85%)	396 (79%)	386 (75%)	0.002*
	Yes	48 (15%)	106 (21%)	126 (25%)	
CKD	No	267 (81%)	441 (88%)	454 (89%)	0.006*
	Yes	61 (19%)	61 (12%)	58 (11%)	
CS	No	281 (86%)	440 (88%)	467 (91%)	0.036*
	Yes	47 (14%)	62 (12%)	45 (9%)	
HT	No	165 (50%)	212 (42%)	202 (39%)	0.007*
	Yes	163 (50%)	290 (58%)	310 (61%)	
ACS	No	286 (87%)	454 (90%)	461 (90%)	0.289
	Yes	42 (13%)	48 (10%)	51 (10%)	
TD	No	262 (80%)	408 (81%)	423 (83%)	0.137
	Hyperthyroidism	31 (9%)	34 (7%)	25 (5%)	
	Hypothyroidism	35 (11%)	60 (12%)	64 (12%)	
TG	<135 mg/dl	261 (82%)	327 (70%)	287 (58%)	<0.001*
	135–200 mg/dl	43 (14%)	109 (23%)	144 (29%)	
	>200 mg/dl	12 (4%)	35 (7%)	66 (13%)	
LDL	<70 mg/dl	65 (21%)	108 (23%)	127 (26%)	0.186
	70–116 mg/dl	143 (45%)	184 (39%)	185 (37%)	
	>116 mg/dl	107 (34%)	177 (38%)	185 (37%)	
HDL	<40 mg/dl	45 (14%)	123 (26%)	154 (31%)	<0.001*
	≥40 mg/dl	270 (86%)	347 (74%)	344 (69%)	
Age (years)	Mean ± SD	71.06 ± 13.45	68.69 ± 13.44	66.47 ± 9.71	<0.001*
	Median	72.00	71.00	68.00	
	Quartiles	65.00–81.00	63.00–78.00	61.00–72.00	
LOHS (days)	Mean ± SD	3.61 ± 3.57	3.40 ± 3.52	3.14 ± 2.90	0.128
	Median	3.00	2.00	2.00	
	Quartiles	2.00–5.00	1.00–4.00	1.00–4.00	
TG (mg/dl)	Mean ± SD	101.54 ± 42.32	121.66 ± 57.16	138.20 ± 70.37	<0.001*
	Median	90.00	109.00	123.00	
	Quartiles	72.00–119.50	84.00–146.00	91.00–166.00	
LDL (mg/dl)	Mean ± SD	105.21 ± 44.37	109.04 ± 49.10	104.27 ± 44.73	0.250
	Median	98.00	97.00	95.00	
	Quartiles	73.00–128.00	72.00–141.00	69.00–132.00	
HDL (mg/dl)	Mean ± SD	53.83 ± 14.66	48.42 ± 12.46	46.33 ± 12.05	<0.001*
	Median	52.00	47.00	45.00	
	Quartiles	43.00–63.00	39.00–57.00	38.00–54.00	
TC (mg/dl)	Mean ± SD	169.21 ± 44.73	167.14 ± 46.75	165.11 ± 44.24	0.448
	Median	160.00	157.00	95.00	
	Quartiles	137.00–198.00	133.00–196.00	69.00–132.00	
CRP (mg/L)	Mean ± SD	5.28 ± 15.93	5.29 ± 14.06	7.13 ± 20.57	0.244
	Median	1.41	1.60	2.02	
	Quartiles	0.74–3.78	0.83–3.45	1.18–5.02	
Albumin (g/dl)	Mean ± SD	3.28 ± 0.70	3.50 ± 0.52	3.57 ± 0.59	0.320
	Median	3.35	3.50	3.80	
	Quartiles	2.85–3.85	3.40–3.70	3.40–4.00	
Transferrin (g/L)	Mean ± SD	2.48 ± 0.71	2.52 ± 0.61	2.65 ± 0.60	0.408
	Median	2.43	2.54	2.52	
	Quartiles	2.04–2.90	2.16–2.88	2.23–3.16	
Lymphocytes (%)	Mean ± SD	23.97 ± 9.87	27.08 ± 9.99	27.96 ± 8.80	0.021*
	Median	23.40	27.25	27.40	
	Quartiles	16.30–31.60	19.10–34.90	22.40–33.70	
PCT (ng/ml)	Mean ± SD	0.72 ± 1.71	0.15 ± 0.30	1.56 ± 8.38	0.435
	Median	0.11	0.06	0.08	
	Quartiles	0.04–0.57	0.02–0.16	0.04–0.22	
BNP (pg/ml)	Mean ± SD	444.29 ± 668.86	331.55 ± 427.12	228.81 ± 287.89	0.003*
	Median	257.40	178.00	143.30	
	Quartiles	141.10–486.60	88.50–391.70	80.30–264.20	
NT-proBNP (pg/ml)	Mean ± SD	3,128.16 ± 6,984.10	2,154.17 ± 4,593.89	1,428.10 ± 2,241.54	0.002*
	Median	1,135.30	925.75	742.90	
	Quartiles	317.40–2,534.50	325.40–2,103.75	314.50–1,544.80	
TSH (uIU/ml)	Mean ± SD	1.69 ± 1.49	1.50 ± 0.98	1.68 ± 1.24	0.014*
	Median	2.98	3.03	2.94	
	Quartiles	2.64–3.23	2.69–3.30	2.56–3.27	
FT4 (ng/dl)	Mean ± SD	1.39 ± 0.33	1.66 ± 2.44	1.41 ± 0.60	0.461
	Median	1.38	1.32	1.33	
	Quartiles	1.15–1.57	1.08–1.49	1.23–1.47	
K (mmol/L)	Mean ± SD	4.37 ± 0.50	4.38 ± 0.45	4.38 ± 0.44	0.998
	Median	4.35	4.39	4.36	
	Quartiles	4.08–4.62	4.10–4.63	4.10–4.61	
Na (mmol/L)	Mean ± SD	139.86 ± 3.00	140.05 ± 3.02	140.22 ± 2.75	0.223
	Median	140.00	140.00	140.00	
	Quartiles	138.00–142.00	139.00–142.00	139.00–142.00	
HbA1c (%)	Mean ± SD	5.95 ± 0.75	6.13 ± 0.85	6.26 ± 0.92	<0.001*
	Median	5.80	6.00	6.10	
	Quartiles	5.50–6.20	5.60–6.40	5.70–6.60	

LOHS, length of hospital stay; N, number of patients; AF, atrial fibrillation; EHRA, European Heart Rhythm Association; NRS, Nutritional Risk Score 2002; BMI, body mass index; HF, heart failure; DM, diabetes mellitus; CKD, chronic kidney disease; CS, cerebral stroke; HT, hypertension; ACS, acute coronary syndrome; TD, thyroid disease; TG, triglycerides; LDL, low-density lipoprotein; HDL, high-density lipoprotein; TC, total cholesterol; CRP, C-reactive protein; PCT, procalcitonin; TSH, thyroid-stimulating hormone; FT3, free triiodothyronine; FT4, free thyroxine; BNP, brain natriuretic peptide; NT-proBNP, N-terminal prohormone of brain natriuretic peptide; K, potassium; Na, sodium; HbA1c, hemoglobin A1c; p, ANOVA test or Kruskal–Wallis test + post hoc analysis (Dunn’s test) for quantitative variables, Chi-squared or Fisher’s exact test for qualitative variables.

*Statistically significant (*p* < 0.05).

### 3.3 Characteristics of the study group by NRS 2002

A comparison of evaluated parameters between groups according to NRS 2002 score is shown in Table 3. Based on the NRS score, two groups were identified: NRS 2002 <3 and ≥3. Statistically significant differences were found according to age, LOHS, BMI, TG, CRP, albumin levels, sex, BMI (categories), CKD, and HT. Study participants with NRS ≥3 were an older group (*M* = 76.3 years), with longer mean LOHS (*M* = 4.44 days), lower BMI (*M* = 24.49 kg/m^2^), lower TG (*M* = 112.2 mg/dl), and albumin levels (*M* = 3.09 g/dl), and higher CRP (*M* = 15.35 mg/dl). This group also had a higher percentage of women (72%), those with a BMI between 18.5 and 24.9 kg/m^2^ (59%), and those with CKD (28%). In addition, a lower percentage of people with HT (44%) was observed in this group.

**TABLE 3:** Comparison of the assessed parameters by NRS 2002.

Parameter		NRS 2002		*P*-value
		
		<3 (*N* = 1,484)	≥3 (*N* = 117)	
Sex	Female	630 (42%)	84 (72%)	<0.001*
	Male	854 (58%)	33 (28%)	
BMI (kg/m^2^)	18.5–24.9	262 (24%)	55 (59%)	<0.001*
	25.0–29.9	416 (37%)	31 (33%)	
	≥30.0	434 (39%)	7 (8%)	
Type of AF	Paroxysmal	584 (39%)	44 (38%)	0.381
	Persistent	622 (42%)	45 (38%)	
	Permanent	278 (19%)	28 (24%)	
EHRA class	I	24 (75)	2 (10%)	0.036
	IIa	114 (26%)	1 (5%)	
	IIb	156 (36%)	3 (14%)	
	III	135 (31%)	14 (67%)	
	IV	8 (2%)	1 (5%)	
HF	No	1,205 (81%)	93 (79%)	0.649
	Yes	279 (19%)	24 (21%)	
DM	No	1,175 (79%)	97 (83%)	0.337
	Yes	309 (21%)	20 (17%)	
CKD	No	1,290 (87%)	84 (72%)	<0.001*
	Yes	194 (13%)	33 (28%)	
CS	No	1,310 (88%)	102 (87%)	0.724
	Yes	174 (12%)	15 (13%)	
HT	No	624 (42%)	65 (56%)	0.005*
	Yes	860 (58%)	52 (44%)	
ACS	No	1,331 (90%)	103 (88%)	0.573
	Yes	153 (10%)	14 (12%)	
TD	No	1,182 (80%)	99 (85%)	0.419
	Hyperthyroidism	107 (7%)	7 (6%)	
	Hypothyroidism	195 (13%)	11 (9%)	
TG	<135 mg/dl	916 (66%)	79 (73%)	0.057
	135–200 mg/dl	341 (25%)	26 (24%)	
	>200 mg/dl	131 (9%)	3 (3%)	
LDL	<70 mg/dl	329 (24%)	22 (20%)	0.547
	70–116 mg/dl	558 (40%)	49 (45%)	
	>116 mg/dl	498 (36%)	37 (35%)	
HDL	<40 mg/dl	354 (26%)	30 (28%)	0.566
	≥40 mg/dl	1,033 (74%)	77 (72%)	
Age (years)	Mean ± SD	68.50 ± 11.98	76.27 ± 13.06	<0.001*
	Median	70.00	79.00	
	Quartiles	63.00–76.00	72.00–85.00	
LOHS (days)	Mean ± SD	3.53 ± 3.36	4.44 ± 4.21	0.005*
	Median	3.00	3.00	
	Quartiles	2.00–5.00	2.00–6.00	
BMI (kg/m^2^)	Mean ± SD	28.89 ± 4.90	24.49 ± 4.21	<0.001*
	Median	28.35	24.00	
	Quartiles	25.20–32.00	21.30–26.80	
TG (mg/dl)	Mean ± SD	124.97 ± 62.26	112.20 ± 40.77	0.036*
	Median	111.00	107.50	
	Quartiles	82.00–152.00	81.50–138.00	
LDL (mg/dl)	Mean ± SD	105.23 ± 45.48	105.06 ± 42.67	0.971
	Median	95.00	97.50	
	Quartiles	70.00–133.00	74.00 + 133.50	
HDL (mg/dl)	Mean ± SD	48.85 ± 13.31	48.13 ± 13.47	0.589
	Median	48.00	46.00	
	Quartiles	39.00–57.00	38.00–58.00	
TC (mg/dl)	Mean ± SD	168.61 ± 46.16	164.64 ± 40.94	0.386
	Median	160.00	156.50	
	Quartiles	135.00–198.00	137.50–188.00	
CRP (mg/L)	Mean ± SD	6.23 ± 18.25	15.35 ± 44.29	<0.001*
	Median	1.76	2.21	
	Quartiles	0.89–4.26	1.02–5.45	
Albumin (g/dl)	Mean ± SD	3.59 ± 0.51	3.09 ± 0.63	0.001*
	Median	3.70	3.30	
	Quartiles	3.40–3.90	2.40–3.50	
Transferrin (g/L)	Mean ± SD	2.59 ± 0.61	2.37 ± 0.74	0.108
	Median	2.53	2.49	
	Quartiles	2.16–2.93	2.01–2.89	
Lymphocytes (%)	Mean ± SD	26.43 ± 9.13	25.40 ± 10.69	0.544
	Median	26.90	24.40	
	Quartiles	19.95–33.10	18.85–32.60	
PCT (ng/ml)	Mean ± SD	0.81 ± 4.81	0.29 ± 0.78	0.618
	Median	0.08	0.08	
	Quartiles	0.03–0.18	0.04–0.17	
TSH (uIU/ml)	Mean ± SD	1.65 ± 1.31	1.74 ± 1.38	0.474
	Median	1.30	1.38	
	Quartiles	0.85–2.07	0.93–2.17	
FT3 (pg/ml)	Mean ± SD	3.02 ± 1.31	2.77 ± 0.69	0.495
	Median	2.92	2.80	
	Quartiles	2.59–3.17	2.26–3.25	
FT4 (ng/dl)	Mean ± SD	1.44 ± 1.25	1.52 ± 0.41	0.826
	Median	1.32	1.46	
	Quartiles	1.13–1.55	1.35–1.71	
BNP (pg/ml)	Mean ± SD	336.91 ± 516.22	407.98 ± 360.56	0.369
	Median	182.45	315.90	
	Quartiles	90.00–361.10	199.60–507.60	
NT-proBNP (pg/ml)	Mean ± SD	2,132.35 ± 3,998.84	5,122.75 ± 10,651.01	<0.001*
	Median	939.00	2,715.90	
	Quartiles	314.20–2,196.40	1,143.00–4,476.10	
K (mmol/L)	Mean ± SD	4.37 ± 0.46	4.38 ± 0.61	0.810
	Median	140.00	140.00	
	Quartiles	139.00–142.00	138.00–142.00	
HbA1c (%)	Mean ± SD	6.16 ± 0.92	6.14 ± 0.85	0.834
	Median	6.00	5.90	
	Quartiles	5.60–6.40	5.60–6.30	

LOHS, length of hospital stay; N, number of patients; AF, atrial fibrillation; EHRA, European Heart Rhythm Association; NRS, Nutritional Risk Score 2002; BMI, body mass index; HF, heart failure; DM, diabetes mellitus; CKD, chronic kidney disease; CS, cerebral stroke; HT, hypertension; ACS, acute coronary syndrome; TD, thyroid disease; TG, triglycerides; LDL, low-density lipoprotein; HDL, high-density lipoprotein; TC, total cholesterol; CRP, C-reactive protein; PCT, procalcitonin; TSH, thyroid-stimulating hormone; FT3, free triiodothyronine; FT4, free thyroxine; BNP, brain natriuretic peptide; NT-proBNP, N-terminal prohormone of brain natriuretic peptide; K, potassium; Na, sodium; HbA1c, hemoglobin A1c; p, t-test or Mann–Whitney U test for quantitative variables, Chi-squared or Fisher’s exact test for qualitative variables.

*Statistically significant (*p* < 0.05).

### 3.4 Length of hospital stay

A comparison of the LOHS according to selected parameters is shown in Table 4. A statistically significant longer length of hospitalization was observed in those with NRS ≥3, persistent AF, EHRA AF Grade II, those without DM, CS, HT, and subjects with LDL levels <70 mg/dl and HDL level <40 mg/dl (Table 4).

**TABLE 4:** Length of hospital stay across groups (qualitative variables): Univariate analysis.

Parameter	Group	Hospitalization (days) [length of stay (days)]			*P*-value
		
		Mean ± SD	Median	Quartiles	
Sex	Female	3.70 ± 3.41	3.00	2.00–5.00	0.066
	Male	3.40 ± 3.40	2.00	2.00–4.00	
NRS	<3	3.53 ± 3.36	3.00	2.00–5.00	0.005*
	≥3	4.44 ± 4.21	3.00	2.00–6.00	
BMI	18.5–24.9	3.61 ± 3.57	3.00	2.00–5.00	0.128
	25.0–29.9	3.40 ± 3.52	2.00	1.00–4.00	
	≥30.0	3.14 ± 2.90	2.00	1.00–4.00	
Type of AF	Paroxysmal	3.34 ± 3.00	3.00	2.00–4.00	< 0.001*
	Persistent	3.38 ± 3.21	2.00	2.00–4.00	
	Permanent	4.28 ± 4.43	3.00	2.00–6.00	
EHRA class	I	2.36 ± 3.94	1.00	0.00–3.00	0.003*
	IIa	3.04 ± 2.38	3.00	2.00–4.00	
	IIb	2.78 ± 1.77	2.00	2.00–4.00	
	III	3.71 ± 2.76	3.00	2.00–5.00	
	IV	3.44 ± 2.83	2.00	2.00–4.00	
HF	No	3.53 ± 3.35	3.00	2.00–5.00	0.935
	Yes	3.54 ± 3.64	3.00	1.00–5.00	
DM	No	3.66 ± 3.53	3.00	2.00–5.00	0.001*
	Yes	3.02 ± 2.87	2.00	1.00–4.00	
CKD	No	3.52 ± 3.37	3.00	2.00–5.00	0.735
	Yes	3.59 ± 3.63	3.00	1.00–5.00	
CS	No	3.67 ± 3.44	3.00	2.00–5.00	< 0.001*
	Yes	2.45 ± 2.93	2.00	0.00–4.00	
HT	No	4.24 ± 3.75	3.00	2.00–6.00	< 0.001*
	Yes	2.99 ± 3.02	2.00	1.00–4.00	
ACS	No	3.58 ± 3.43	3.00	2.00–5.00	0.078
	Yes	3.11 ± 3.17	2.00	1.00–4.00	
TD	No	3.48 ± 3.38	3.00	2.00–5.00	0.418
	Hyperthyroidism	3.82 ± 3.24	3.00	2.00–5.00	
	Hypothyroidism	3.70 ± 3.66	2.00	1.00–5.00	
TG	<135 mg/dl	3.60 ± 3.50	3.00	2.00–5.00	0.865
	135–200 mg/dl	3.57 ± 3.38	3.00	2.00–5.00	
	>200 mg/dl	3.75 ± 2.68	3.00	2.00–5.00	
LDL	<70 mg/dl	4.10 ± 3.85	3.00	2.00–6.00	< 0.001*
	70–116 mg/dl	4.00 ± 3.67	3.00	2.00–5.00	
	>116 mg/dl	2.85 ± 2.52	2.00	1.00–4.00	
HDL	<40 mg/dl	4.52 ± 4.21	3.50	2.00–6.00	< 0.001*
	≥40 mg/dl	3.28 ± 2.98	2.00	0.00–3.00	

LOHS, length of hospital stay; N, number of patients; AF, atrial fibrillation; EHRA, European Heart Rhythm Association; NRS, Nutritional Risk Score 2002; BMI, body mass index; HF, heart failure; DM, diabetes mellitus; CKD, chronic kidney disease; CS, cerebral stroke; HT, hypertension; ACS, acute coronary syndrome; TD, thyroid disease; TG, triglycerides; LDL, low-density lipoprotein; HDL, high-density lipoprotein; 2-group comparison, *p*, *t*-test or Mann–Whitney U test; >2-group comparison, ANOVA test or Kruskal–Wallis test + *post hoc* analysis (Dunn’s test).

*Statistically significant (*p* < 0.05).

Table 5 shows the assessment of the effect of selected parameters on the LOHS (days) (univariate model of predictors included in the analysis). The variables included in the analysis were age (years), NRS (points, as a quantitative and qualitative variable ref. <3), BMI (kg/m^2^, as a quantitative and qualitative variable ref. 18.5–24.9), TG (mg/dl), LDL (mg/dl, as a quantitative and qualitative variable ref. <70 mg/dl), HDL (mg/dl, as a quantitative and qualitative variable ref. <40 mg/dl), TC (mg/dl, as a quantitative as well as qualitative variable ref. <135 mg/dl), CRP (mg/L), albumin (g/dl), transferrin (g/L), lymphocytes (%), PCT (ng/ml), TSH (uIU/ml), FT3 (pg/ml), FT4 (ng/dl), BNP (pg/ml), K (mmol/L), Na (mmol/L), HbA1c (%), sex (ref. male), type of AF (ref. permanent), EHRA AF (ref. I), HF, DM, CKD, CS, HT, ACS, and thyroid disease (TD). Linear regression analysis in the univariate model showed the effect of age (*B* = 0.04, *p* < 0.001), LDL (*B* = −0.01, *p* < 0.001), HDL (*B* = −0.04, *p* < 0.001), TC (*B* = −0.01, *p* < 0.001), CRP (*B* = 0.03, *p* < 0.001), transferrin (*B* = −1.23, *p* = 0.032), lymphocytes (*B* = −0.09, *p* < 0.001), BNP (*B* = 0.00, *p* < 0.001), Na (*B* = −0.13, *p* < 0.001), and HbA1c (*B* = 0.27, *p* = 0.015) on LOHS. In addition, factors such as NRS (ref. <3, *B* = 0.46, *p* = 0.005), type of AF (ref. permanent, paroxysmal: *B* = −0.33, *p* = 0.004, persistent: *B* = −0.28, *p* = 0.010), DM (ref. no; *B* = −0.32, *p* = 0.001), CS (ref. no; *B* = −0.61, *p* < 0.001), HT (ref. no; *B* = −0.63, *p* < 0.001), LDL (ref. <70; 70–116 mg/dl: *B* = 0.35, *p* < 0.002; >116 mg/dl: *B* = −0.90, *p* < 0.001), and HDL (ref. <40 mg/dl; *B* = −0.62, *p* < 0.001) had an impact. Variables included in the multivariate model were age, LDL (mg/dl), HDL (mg/dl), Na, K, presence of CS, and HT (Table 5).

**TABLE 5:** Effect of selected parameters on length of hospital stay (days) (univariate model and multivariate predictors included in the linear regression analysis).

Parameter		Unadjusted model					Adjusted model				
			
		*B*	SE	*t*	*P*-value	β	*B*	SE	*t*	*P*-value	β
Age (years)		0.04	0.01	6.60	0.000	0.15	0.04	0.01	5.77	0.000	0.17
NRS (points)		–0.06	0.09	–0.64	0.519	–0.02	−	−	−	−	−
BMI (kg/m^2^)		–0.03	0.02	–1.46	0.146	–0.04	−	−	−	−	−
TG (mg/dl)		0.00	0.00	0.60	0.551	0.01	−	−	−	−	−
LDL (mg/dl)		–0.01	0.00	–7.40	0.000	–0.18	–0.01	0.00	–2.78	0.006	–0.08
HDL (mg/dl)		–0.04	0.01	–7.04	0.000	–0.17	–0.04	0.01	–5.06	0.000	–0.15
TC (mg/dl)		–0.01	0.00	–4.05	0.000	–0.10	−	−	−	−	−
CRP (mg/L)		0.03	0.00	7.56	0.000	0.20	−	−	−	−	−
Albumin (g/dl)		–0.55	1.04	–0.53	0.596	–0.06	−	−	−	−	−
Transferrin (g/L)		–1.23	0.57	–2.16	0.032	–0.16	−	−	−	−	−
Lymphocytes (%)		–0.09	0.02	–4.46	0.000	–0.21	−	−	−	−	−
PCT (ng/ml)		0.11	0.10	1.08	0.282	0.09	−	−	−	−	−
TSH (uIU/ml)		0.09	0.06	1.35	0.177	0.03	−	−	−	−	−
FT3 (pg/ml)		–0.06	0.28	–0.20	0.841	–0.01	−	−	−	−	−
FT4 (ng/dl)		0.02	0.28	0.08	0.940	0.01	−	−	−	−	−
BNP (pg/ml)		0.00	0.00	3.88	0.000	0.17	−	−	−	−	−
NT-proBNP (pg/ml)		0.00	0.00	5.37	0.000	0.19	−	−	−	−	−
K (mmol/L)		–0.33	0.17	–1.94	0.053	–0.05	–0.45	0.20	–2.19	0.028	–0.06
Na (mmol/L)		–0.13	0.03	–5.14	0.000	–0.12	–0.14	0.03	–4.34	0.000	–0.12
HbA1c (%)		0.27	0.11	2.43	0.015	0.07	−	−	−	−	−
Sex (ref. male)	Female	0.15	0.08	1.84	0.066	0.04	−	−	−	−	−
NRS (ref. <3)	≥3	0.46	0.16	2.79	0.005	0.07	−	−	−	−	−
BMI (kg/m^2^) (ref. 18.5–24.9)	25.0—29.9	0.02	0.13	0.13	0.893	0.00	−	−	−	−	−
	≥30.0	–0.24	0.13	–1.93	0.054	–0.06	−	−	−	−	−
Type of AF (ref. permanent)	Paroxysmal	–0.33	0.11	–2.88	0.004	–0.07	−	−	−	−	−
	Persistent	–0.28	0.11	–2.56	0.010	–0.06	−	−	−	−	−
EHRA class (ref. I)	IIa	–0.03	0.26	–1.15	0.249	–0.01	−	−	−	−	−
	IIb	–0.29	0.28	–1.04	0.298	–0.07	−	−	−	−	−
	III	0.63	0.28	2.26	0.024	0.16	−	−	−	−	−
	IV	0.37	0.64	0.56	0.564	0.47	−	−	−	−	−
HF	Yes	0.01	0.10	0.08	0.935	0.00	−	−	−	−	−
DM	Yes	–0.32	0.10	–3.27	0.001	–0.08	−	−	−	−	−
CKD	Yes	0.04	0.12	0.30	0.765	0.01	−	−	−	−	−
CS	Yes	–0.61	0.13	–4.85	0.000	–0.11	–0.49	0.15	–3.27	0.001	–0.09
HT	Yes	–0.63	0.08	–7.88	0.000	–0.18	–0.52	0.10	–5.37	0.000	–0.16
ACS	Yes	–0.23	0.13	–1.76	0.078	–0.04	−	−	−	−	−
Thyroid disease	Hyperthyroidism	0.15	0.22	0.70	0.487	0.03	−	−	−	−	−
	Hypothyroidism	0.03	0.18	0.18	0.861	0.01	−	−	−	−	−
TG (ref. <135 mg/dl)	135–200 mg/dl	–0.07	0.15	–0.45	0.655	–0.02	−	−	−	−	−
	>200 mg/dl	0.10	0.19	0.54	0.592	0.02	−	−	−	−	−
LDL (ref. <70 mg/dl)	70–116 mg/dl	0.35	0.11	3.16	0.002	0.08	−	−	−	−	−
	>116 mg/dl	–0.80	0.11	–7.08	0.000	–0.18	−	−	−	−	−
HDL (ref. <40 mg/dl)	≥40 mg/dl	–0.62	0.09	–6.72	0.000	–0.16	−	−	−	−	−

LOHS, length of hospital stay; N, number of patients; AF, atrial fibrillation; EHRA, European Heart Rhythm Association; NRS, Nutritional Risk Score 2002; BMI, body mass index; HF, heart failure; DM, diabetes mellitus; CKD, chronic kidney disease; CS, cerebral stroke; HT, hypertension; ACS, acute coronary syndrome; TD, thyroid disease; TG, triglycerides; LDL, low-density lipoprotein; HDL, high-density lipoprotein; TC, total cholesterol; CRP, C-reactive protein; PCT, procalcitonin; TSH, thyroid-stimulating hormone; FT3, free triiodothyronine; FT4, free thyroxine; BNP, brain natriuretic peptide; NT-proBNP, N-terminal prohormone of brain natriuretic peptide; K, potassium; Na, sodium; HbA1c, hemoglobin A1c; B, unstandardized regression coefficient *B*; SE, standard error; *t*, *B*/standard error; β, standardized regression coefficient β.

Table 6 shows the evaluation of the effect of selected parameters on the LOHS (<5 vs. ≥5 days) (univariate and multivariate models of the predictors included in the logistic regression analysis). The variables included in the analysis were age (years), NRS (points, as a quantitative and qualitative variable ref. <3), BMI (kg/m^2^, as a quantitative and qualitative variable ref. 18.5–24.9), TG (mg/dl), LDL (mg/dl, as a quantitative and qualitative variable ref. <70 mg/dl), HDL (mg/dl, as a quantitative and qualitative variable ref. <40 mg/dl), TC (mg/dl, as a quantitative and qualitative variable ref. <135 mg/dl), CRP (mg/L), albumin (g/dl), transferrin (g/L), lymphocytes (%), PCT (ng/ml), TSH (uIU/ml), FT3 (pg/ml), FT4 (ng/dl), BNP (pg/ml), K (mmol/L), Na (mmol/L), HbA1c (%), sex (ref. male), type of AF (ref. permanent), EHRA AF (ref. I), HF, DM, CKD, CS, HT, ACS, and TD. Logistic regression analysis in the univariate model showed the effect of age (OR = 1.04, *p* < 0.001), LDL (OR = 0.99, *p* < 0.001), HDL (OR = 0.98, *p* < 0.001), TC (OR = 0.996, *p* < 0.001), CRP (OR = 1, 02, *p* < 0.001), lymphocytes (OR = 0.97, *p* = 0.008), BNP (OR = 0.00, *p* < 0.001), K (OR = 0.70, *p* = 0.002), Na (OR = 0.93, *p* < 0.001), and HbA1c (OR = 1.16, *p* = 0.027) on LOHS. In addition, factors such as sex (ref. male, OR = 1.34, *p* = 0.006), NRS (ref. <3, OR = 1.55, *p* = 0.029), type of AF (ref. permanent, paroxysmal: OR = 0.57, *p* < 0.001, persistent: OR = 0.57, ≤0.001), DM (ref. no; OR = 0.75, *p* = 0.038), CS (ref. no; OR = 0.55, *p* = 0.002), HT (ref. no; OR = 0.50, *p* < 0.001), TD (ref. no; hyperthyroidism: OR = 1.50, *p* = 0.039), LDL (ref. <70; >116 mg/dl: OR = 0.49, *p* < 0.001), and HDL (ref. <40 mg/dl; OR = 0.56, *p* < 0.001) had an impact on LOHS. The age, HDL (mg/dl), and HT variables were included in the multivariate model (Table 6).

**TABLE 6:** Effect of selected parameters on length of hospital stay (<5 vs. ≥5 days) (univariate model and multivariate predictors included in logistic regression analysis).

Parameter		Unadjusted model					Adjusted model				
			
		*B*	*P*-value	OR	−95% CI	+95% CI	*B*	*P*-value	OR	−95% CI	+95% CI
Age (years)		0.04	0.000	1.04	1.03	1.05	0.04	0.000	1.04	1.03	1.05
NRS (points)		0.09	0.122	1.09	0.98	1.23	−	−	−	−	−
BMI (kg/m^2^)		0.00	0.706	1.00	0.97	1.02	−	−	−	−	−
TG (mg/dl)		0.00	0.818	1.00	1.00	1.00	−	−	−	−	−
LDL (mg/dl)		–0.01	0.000	0.99	0.99	0.99	−	−	−	−	−
HDL (mg/dl)		–0.02	0.000	0.98	0.97	0.99	–0.03	0.000	0.97	0.96	0.98
TC (mg/dl)		0.00	0.001	0.996	0.994	0.998	−	−	−	−	−
CRP (mg/L)		0.02	0.000	1.02	1.01	1.02	−	−	−	−	−
Albumin (g/dl)		0.52	0.162	1.68	0.81	3.47	−	−	−	−	−
Transferrin (g/L)		–0.13	0.596	0.88	0.55	1.41	−	−	−	−	−
Lymphocytes (%)		–0.03	0.008	0.97	0.95	0.99	−	−	−	−	−
PCT (ng/ml)		0.95	0.128	2.58	0.76	8.78	−	−	−	−	−
TSH (uIU/ml)		0.06	0.109	1.07	0.99	1.15	−	−	−	−	−
FT3 (pg/ml)		0.07	0.550	1.08	0.85	1.37	−	−	−	−	−
FT4 (ng/dl)		–0.07	0.633	0.93	0.70	1.25	−	−	−	−	−
BNP (pg/ml)		0.00	0.021	1.00	1.00	1.00	−	−	−	−	−
NT-proBNP (pg/ml)		0.00	<0.001	1.00	1.00	1.00	−	−	−	−	−
K (mmol/L)		–0.35	0.002	0.70	0.56	0.88	−	−	−	−	−
Na (mmol/L)		–0.08	0.000	0.93	0.90	0.96	−	−	−	−	−
HbA1c (%)		0.15	0.027	1.16	1.02	1.32	−	−	−	−	−
Sex (ref. male)	Female	0.29	0.006	1.34	1.09	1.65	−	−	−	−	−
NRS (ref. <3)	≥3	0.44	0.029	1.55	1.05	2.31	−	−	−	−	−
BMI (kg/m^2^) (ref. 18.5–24.9)	25.0—29.9	–0.16	0.311	0.85	0.62	1.17	−	−	−	−	−
	≥30.0	–0.17	0.296	0.85	0.62	1.16	−	−	−	−	−
Type of AF (ref. permanent)	Paroxysmal	–0.56	0.000	0.57	0.43	0.76	−	−	−	−	−
	Persistent	–0.55	0.000	0.57	0.44	0.76	−	−	−	−	−
EHRA class (ref. I)	IIa	0.77	0.175	2.17	0.71	6.62	−	−	−	−	−
	IIb	0.28	0.961	1.03	0.33	3.22	−	−	−	−	−
	III	1.05	0.062	2.84	0.95	8.51	−	−	−	−	−
	IV	0.73	0.450	2.07	0.31	13.77	−	−	−	−	−
HF	Yes	0.01	0.930	1.01	0.78	1.32	−	−	−	−	−
DM	Yes	–0.28	0.038	0.75	0.58	0.98	−	−	−	−	−
CKD	Yes	0.24	0.115	1.27	0.94	1.70	−	−	−	−	−
CS	Yes	–0.60	0.002	0.55	0.38	0.80	−	−	−	−	−
HT	Yes	–0.69	0.000	0.50	0.41	0.62	–0.55	0.000	0.58	0.44	0.76
ACS	Yes	–0.16	0.368	0.85	0.60	1.21	−	−	−	−	−
Thyroid disease	Hyperthyroidism	0.41	0.039	1.50	1.02	2.21	−	−	−	−	−
	Hypothyroidism	0.20	0.195	1.23	0.90	1.67	−	−	−	−	−
TG (ref. <135 mg/dl)	135–200 mg/dl	–0.08	0.517	0.92	0.71	1.18	−	−	−	−	−
	>200 mg/dl	0.00	1.000	1.00	0.69	1.46	−	−	−	−	−
LDL (ref. <70 mg/dl)	70–116 mg/dl	–0.07	0.581	0.93	0.71	1.21	−	−	−	−	−
	>116 mg/dl	–0.72	0.000	0.49	0.37	0.65	−	−	−	−	−
HDL (ref. <40 mg/dl)	≥40 mg/dl	–0.58	0.000	0.56	0.45	0.71	−	−	−	−	−

LOHS, length of hospital stay; N, number of patients; AF, atrial fibrillation; EHRA, European Heart Rhythm Association; NRS, Nutritional Risk Score 2002; BMI, body mass index; HF, heart failure; DM, diabetes mellitus; CKD, chronic kidney disease; CS, cerebral stroke; HT, hypertension; ACS, acute coronary syndrome; TD, thyroid disease; TG, triglycerides; LDL, low-density lipoprotein; HDL, high-density lipoprotein; TC, total cholesterol; CRP, C-reactive protein; PCT, procalcitonin; TSH, thyroid-stimulating hormone; FT3, free triiodothyronine; FT4, free thyroxine; BNP, brain natriuretic peptide; NT-proBNP, N-terminal prohormone of brain natriuretic peptide; K, potassium; Na, sodium; HbA1c, hemoglobin A1c; B, regression coefficient *B*; OR, odds ratio; CI, confidence intervals.

## 4 Discussion

Abnormal nutritional status is a common problem in hospitalized patients and can affect up to 30%. In hospitalized patients, it is associated with a prolonged hospital stay, increased risk of re-hospitalization, hospital infections, medical treatment complications, and thus increased hospital costs (19–21). Poor nutritional status in CVD, such as ACS and HF, has also been shown to be associated with a higher risk of in-hospital death (22, 23). In our sample, the average LOHS in patients with AF was 3.5 days. This result is consistent with the observations of other researchers, where it was 3–4 days (24, 25). Unfortunately, nutritional status, especially malnutrition, is often ignored in clinical practice, and many patients with CVD are not properly diagnosed, which can result in a worsened disease course. Malnutrition in patients with CVD is particularly problematic, as in many situations it can even lead to cardiac cachexia, which can worsen the course of the underlying disease, creating a vicious cycle. It is also worth noting that patients with CVD typically take multiple medications, and polypharmacy is another element that can affect nutritional status. Medications, among other things, can interfere with the absorption of nutrients and even disrupt the sense of taste (26).

In this study, patients at risk of malnutrition stayed in the hospital significantly longer (Me: 4.44 days). They also constituted a larger group of patients hospitalized for longer than 5 days than those hospitalized for less than 5 days. Malnutrition more often affected women, and the mean age of patients affected was 76 ± 13 years. Additionally, the univariate linear analysis showed that the risk of malnutrition, according to the NRS 2002, was associated with more extended hospitalization (by 0.46 days). The univariate logistic regression model showed that the risk of hospitalization for longer than 5 days was 55% higher in these patients. Not many papers evaluate the effect of nutritional status on the LOHS of patients with AF. Regarding length in general, the study by Guha et al. showed that the LOHS in the case of primary AF was 1.9 ± 0.002 and in the case of concomitant cancer was 2.9 ± 0.1 days. A significantly longer stay was characterized in this study by the group of patients who underwent direct current cardioversion (27). In the Vijan et al. study, independent factors affecting LOHS (≥7 days for this study) included ACS, decompensated HF, increased NT-proBNP levels and infections (25). Researchers studying nutritional status confirm that malnutrition upon hospital admission is quite common and is associated not only with prolonged hospital stay but also with worse prognosis (28–31). Malnutrition also increases the risk of re-hospitalization in patients with AF. In Budzyński and Anaszewicz’s study, the average LOHS was 3.7 days, and patients with NRS 2002 ≥3 accounted for 3.6% of patients. In our group of patients, the LOHS was similar, NRS 2002 ≥3 represented 7% of patients. The patients’ BMIs were also similar. In both our study and that of Budzynski et al., 75% of patients struggled with overweight and obesity. Both studies were conducted in Poland (32). BMI is not a perfect tool for assessing nutritional status because it does not assess individual components of body weight. Patients with multimorbidity, such as those with HF or CKD, may have edema, which overestimates body mass (33, 34). For overweight individuals, an increase in the heart’s minute volume contributes to remodeling of the heart’s structure, which is the basis for this cardiac arrhythmia (35). In addition, chronic inflammation typical of both obesity and malnutrition is a significant factor strongly associated with the occurrence of AF (36). In malnutrition, inflammation leads to increased oxidative stress and thus endothelial dysfunction, which can lead to serious cardiovascular and cerebrovascular events (37). In a study by Zhu et al., patients who are malnourished after ablation are at a higher risk of AF recurrence, which may be associated with both longer hospital stays and re-hospitalization (38).

Although in our study, BMI scores were not a factor in the LOHS, it should be noted that many publications show a positive correlation between the occurrence of AF and underweight, overweight and obesity (39–41). The authors also emphasize that obesity, as measured by BMI, is associated with increased morbidity and death from various causes (42).

In this study, in the multivariate linear regression model, the factors influencing shorter LOHS were higher LDL and HDL levels, and in the multivariate logistic regression model, the risk of hospitalization for longer than 5 days was lower for higher HDL levels. As mentioned earlier, in the study group, 75% of patients suffered from overweight and obesity. Bora et al. showed that 79.8% of the decrease in HDL in the study population was associated with an increase in BMI in those who were overweight or obese (43). These patients may also have been previously diagnosed with CVD risk, which may have influenced the earlier initiation of treatment and, for example, the recommendation to take LDL-lowering drugs (44). The study by Barkas et al. found that patients with AF had lower LDL and HDL levels than patients without AF. The researchers also found that low HDL levels may be a predictor of AF (45). According to Suzuki, both older age and a decrease in blood lipids can cause AF due to abnormal SA node conduction, left atrial enlargement, and myocardial degeneration (46). In the case of coexisting hyperthyroidism, LDL concentrations will also be lower. Thyroxine induces the activity of 3-hydroxy-3-methylglutaryl coenzyme and what is associated with this is a reduction in LDL concentrations (47). In the study group, hyperthyroidism affected 7% of patients. The low LDL and HDL levels could also have been caused by malnutrition (48). However, in the study group, they occurred in only 7% of the subjects. The occurrence of AF is also influenced by inflammation and oxidative stress, which occurs when HDL levels are low (49). Numerous studies show that patients taking lipid-lowering drugs have a significantly reduced risk of AF (50, 51). Lee et al. showed that lower cholesterol levels were associated with a higher risk of incident AF and cholesterol variability with AF development. Their findings confirm the occurrence of the “cholesterol paradox” (52).

In this study, higher potassium and sodium concentrations were also associated with shorter hospitalization. Studies by other authors confirm that lower potassium concentrations in patients with atrial arrhythmias are associated with an increased risk of AF (53–55). Hyponatremia is also independently associated with the occurrence of AF. Low potassium and sodium levels-induced slowing of sinoatrial node beating rate and genesis of pulmonary vein burst firing which could contribute to the higher occurrence of AF during hyponatremia or hypokalemia. Electrolyte disturbances play an important role in the pathogenesis of AF and they too may be related to malnutrition (56–58).

The study had its strengths and weaknesses. Undoubtedly, the strengths included the size of the study group. One of the limitations of the study was the small number of malnourished patients. They accounted for 7% of the study group. Sometimes the NRS 2002 or BMI score was missing from the patient’s medical records. Waist-Hip Ratio measurement or electrical bioelectrical impedance analysis was not performed on admission to the hospital. Medical records also lacked information on patients’ previous treatment, such as drugs to reduce lipids.

## 5 Conclusion

For nutritional status, factors indicating the risk of prolonged hospitalization in patients with AF are malnutrition, lower serum LDL, HDL, potassium, and sodium levels determined at the time of admission to the cardiology department. Assessment of nutritional status in patients with AF is important both in the context of evaluating obesity and malnutrition status, as both conditions can alter the prognosis of patients. Further studies are needed to determine the exact impact of the above on the risk of prolonged hospitalization.

## Data availability statement

The original contributions presented in this study are included in the article/supplementary material, further inquiries can be directed to the corresponding author.

## Ethics statement

The studies involving human participants were reviewed and approved by the Independent Bioethics Committee of Wroclaw Medical University, protocol no. KB-205/2021. Written informed consent for participation was not required for this study in accordance with the national legislation and the institutional requirements.

## Author contributions

MC, IU, and RJ-V: conceptualization and formal analysis. MC, IU, RJ-V, and JS: methodology. IU, AD, and MK-O: software. MC, IU, RJ-V, JS, MK-O, and AD: investigation and writing—original draft preparation. MC, KŁ, RJ-V, and RB-T: writing—review and editing. MC, KŁ, AD, and RB-T: visualization. JS: supervision. MC and IU: project administration. All authors read and agreed to the published version of the manuscript.
